# Eriodictyol Inhibits Proliferation, Metastasis and Induces Apoptosis of Glioma Cells *via* PI3K/Akt/NF-κB Signaling Pathway

**DOI:** 10.3389/fphar.2020.00114

**Published:** 2020-02-25

**Authors:** Wenjun Li, Qian Du, Xiaoli Li, Xiangru Zheng, Feng Lv, Xin Xi, Guili Huang, Jia Yang, Songqing Liu

**Affiliations:** ^1^ Department of Pharmacy, The Third Affiliated Hospital of Chongqing Medical University (Gener Hospital), Chongqing, China; ^2^ College of Pharmacy, Chongqing Medical University, Chongqing, China; ^3^ Department of Gastrointestinal Surgery, The First Affiliated Hospital of Chongqing Medical University, Chongqing, China

**Keywords:** eriodictyol, glioma, apoptosis, PI3K, NF-κB

## Abstract

Glioma is the most common type of malignant brain tumor. Due to its highly aggressive and metastatic features, glioma is associated with poor prognosis and a lack of effective treatments. Eriodictyol, a natural flavonoid compound, has been reported to possess anti-inflammatory and antioxidant effects. However, the anti-tumor effects of eriodictyol and the underlying mechanisms have rarely been reported. In this study, we found that eriodictyol has anti-tumor activity in lung, colon, breast, pancreas, and liver cancer, and most significantly in glioma cell lines. Eriodictyol dose- and time-dependently suppresses cell proliferation, migration, and invasion in U87MG and CHG-5 glioma cells. In addition, eriodictyol induces apoptosis in U87MG and CHG-5 cells, as evaluated by flow cytometry, immunofluorescence, and Western blot. Furthermore, eriodictyol downregulates the phosphoinositide 3-kinase (PI3K)/Akt/NF-κB signaling pathway in a concentration-dependent manner. Moreover, the effects of eriodictyol on the apoptosis of glioma cells are enhanced by LY294002 (a PI3K inhibitor) and reversed by 740 Y-P (a PI3K agonist). In a mouse xenograft model, eriodictyol not only dramatically suppressed tumor growth but also induced apoptosis in tumor cells. In summary, our data illustrate that eriodictyol effectively inhibits proliferation and metastasis and induces apoptosis of glioma cell lines, which might be a result of the blockade of the PI3K/Akt/NF-κB signaling pathway.

## Introduction

Glioma is the most prevalent type of malignant tumor in the central nervous system (CNS), and it represents 75% of malignant brain tumors (Lapointe et al., 2018). Due to its highly aggressive and metastatic features, even if glioma patients are treated with the “golden standard” strategies including surgery, radiotherapy, and chemotherapy, the prognosis remains poor (Stupp et al., 2005). According to an American survey, the 5-year survival rate of glioma patients is only 5% (Alexander and Cloughesy, 2017). Temozolomide (TMZ) is the most common drug used as glioma therapy, but the efficacy is often limited because of the side effects and the development of resistance. Therefore, it is particularly urgent to find new highly efficient and less-toxic anti-glioma drugs.

**Figure 1:**
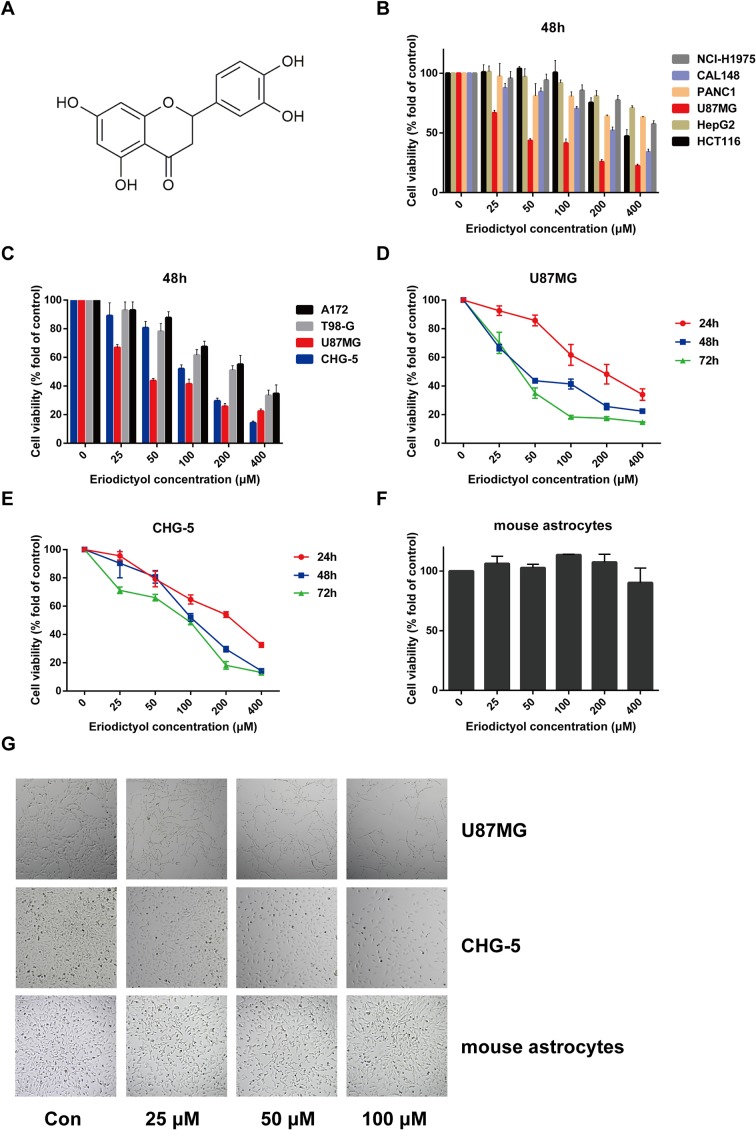
Eriodictyol suppresses the proliferation of cancer cell lines *in vitro*. **(A)** The structure of eriodictyol. **(B)** Anti-cancer activity of eriodictyol. Several cancer cell lines were treated with different concentrations of eriodictyol (0, 25, 50, 100, 200, or 400 μM) for 48 h, and cell viability was measured by CCK-8 assay. **(C)** The treatment of four different glioma cell lines gave results similar to those presented in **(B)**. **(D**, **E)** Glioma cell lines U87MG and CHG-5 were treated with varying concentrations of eriodictyol for 24, 48, or 72 h, and subjected to CCK-8 assay. **(F)** Cytotoxic effects of eriodictyol. Normal mouse astrocytes were treated with different concentrations of eriodictyol for 48 h, and cell viability was evaluated by CCK-8 assay. **(G)** U87MG cells, CHG-5 cells, and normal mouse astrocytes were treated with different concentrations of eriodictyol for 48 h, and then the cells were harvested for macrophage observation at 40× magnification. Data are presented as the mean ± SD of three experiments.

In recent years, natural compounds have become a hot research topic in preventing and treating cancer, due to their potential multiple targets and bioactivities and their limited toxicity (Efferth et al., 2019). Eriodictyol (**Figure 1A**), a natural flavonoid compound, is ubiquitous in fruits, vegetables, and several Chinese medicines (Minato et al., 2003; Zeng et al., 2016). In addition, it has been reported that eriodictyol exerts anti-inflammatory, antioxidant, and neuroprotective effects (He et al., 2018; Kwon and Choi, 2019; Lv et al., 2019). In *in vitro* studies, scientists have found that eriodictyol exerts its anti-inflammatory and antioxidant effects through Akt- and NF-κB-related signaling pathways (Xie et al., 2017; Liu and Yan, 2019). However, the anti-cancer activity of eriodictyol and its underlying mechanisms have been less explored. Ahmad et al. reported that the Akt/NF-κB signaling pathway plays a very important role in the development of cancers (Ahmad et al., 2013). Thus, we hypothesized that eriodictyol might have anti-tumor effects.

In this study, we focused on the activity of eriodictyol in cancers. Our findings indicate that eriodictyol has anti-tumor activity in lung, colon, breast, pancreas, liver cancer, and in glioma cell lines especially. Eriodictyol dramatically inhibits glioma cell growth, migration, invasion and induces apoptosis by blocking the PI3K/Akt/NF-κB signaling pathway. Moreover, the anti-glioma activities of eriodictyol were evaluated using a xenograft mouse model. We found that eriodictyol can inhibit tumor growth in nude mice by suppressing proliferation and enhancing apoptosis. Therefore, we conclude that eriodictyol might be used as a therapeutic agent against glioma.

## Materials and Methods

### Reagents

Temozolomide (TMZ) and eriodictyol (purity ≥ 98%) were purchased from Dalian Meilun Biotechnology, Co., Ltd. (Dalian, China) and dissolved in dimethyl sulfoxide (DMSO). The final concentration of DMSO in culture medium was ≤ 1 ‰. LY294002 and 740 Y-P were bought from MedChemExpress (MCE, United States). Hoechst 33342, the TUNEL apoptosis assay kit, and Lipo8000 were obtained from Beyotime Biotechnology, Co., Ltd. (Shanghai, China). The Annexin V–FITC/PI Apoptosis Detection Kit was purchased from Vazyme Biotech, Co., Ltd. (Nanjing, China). Antibodies against PI3K, phospho-PI3K (Y607), and Caspase-9 and goat anti-rabbit IgG H&L (IRDye 800CW) pre-adsorbed secondary antibodies were obtained from Abcam (Cambridge, United Kingdom). Antibodies against Akt, phospho-Akt (S473), Caspase-3, cleaved Caspase-3, cleaved Caspase-8, Bax, Bcl-2, Bcl-xL, and NF-κB were bought from Cell Signaling Technology (Danvers, MA, United States). Antibodies against Ki67, phospho-NF-κB (S536) and phospho-IκBα (S32/S36) were obtained from Affinity Biosciences (Zhenjiang, China). Antibodies against IκBα and PARP were purchased from ProteinTech Group, Inc. (Chicago, IL, United States). Antibodies against β-actin and the Cell Counting Kit-8 (CCK-8) were purchased from Bimake (Houston, TX, United States).

### Cell Culture

Human glioma cell lines U87MG, A172, T98-G, the non-small cell lung cancer cell line NCI-H1975, the colorectal cancer cell line HCT116, the hepatoma cell line HepG2, and the pancreatic cancer cell line PANC1 were obtained from the cell bank of the Chinese Academy of Sciences (Shanghai, China). The human breast cancer cell line CAL148, the glioma cell line CHG-5, and primary mouse astrocytes were a gift from Associate Professor Li Xiaoli (College of Pharmacy, Chongqing Medical University, Chongqing, China). All cells were cultured in DMEM medium with 10% fetal bovine serum (FBS, Biological Industries, Kibbutz Beit-Haemek, Israel) and 1% penicillin/streptomycin in a humidified atmosphere of 5% CO_2_ at 37°C.

### Cell Viability Assay

The effects of eriodictyol on viability were assessed using the CCK-8 assay. Cells were plated at 3 ×10^3^ cells per well in 96-well plates. On the following day, 0–400 μM eriodictyol was added to the medium. After 24–72 h, cells were incubated with 10 μl CCK-8 for 2 h at 37°C with 5% CO_2_. Absorbance was measured at 450 nm using a SynergyH1 microplate spectrophotometer (BioTek).

### Colony Formation Assay

U87MG and CHG-5 cells were plated in six-well plates (500 cells/well). After the cells were allowed to attach for 12 h, the cells were treated with different concentrations (0, 10, 20, 40 or μM) of eriodictyol, and the medium was changed every 3 days. After 12 days, the medium was discharged. The cells were washed with cold PBS, fixed in 4% paraformaldehyde for 30 min, and stained with 0.5% crystal violet solution for 15 min. After washing away excessive crystal violet and drying, the colonies were observed, and the results were analyzed using Image J software.

### Wound Healing Assay

U87MG and CHG-5 cells were plated in six-well plates with DMEM containing 10% FBS. After the cells had 100% conﬂuent, a 200 μl pipette tip was used to scrape the wells to mimic wounds. The cells were gently washed twice with PBS to remove the detached cells and cultured in DMEM containing 2% FBS. The cells were treated with various concentrations (0, 25, 50, 100 μM) of eriodictyol. After 0, 12, and 24 h, the cells were photographed using a light microscope (Nikon, Japan) and the area of the wound was measured.

### Transwell Migration Assay

Transwell chambers (8-μm pore size; 24-well) were used for migration assays (Costar; Corning Incorporated, Corning, NY, United States). U87MG and CHG-5 cells were plated into the upper chamber at 1 × 10^4^ cells/well in 200 μl of serum-free DMEM, while the lower chamber was filled with DMEM containing 10% FBS. Various concentrations (0, 25, 50, 100 μM) of eriodictyol were added to the upper chambers. After 24 h incubation at 37°C, non-migrated cells were removed from the upper side of each chamber with a cotton swab. Cells on the lower side of the chamber were fixed with 4% paraformaldehyde for 30 min at room temperature and then stained with 0.5% crystal violet for 15 min. Migrated cells were counted at 100× magnification using a microscope (Nikon).

### Transwell Invasion Assay

The Transwell chamber was pretreated with Matrigel (Corning, NY, United States) and dried at 37°C for 1 h. Other procedures were the same as for the Transwell migration assay. The results of the Transwell invasion assay were also calculated according to the number of transferred cells.

### Cell Cycle Analysis

U87MG and CHG-5 cells were plated in six-well plates at a density of 2 × 10^5^ cells per well. After 12 h, various concentrations (0, 25, 50, 100 μM) of eriodictyol were added to each well, and cells were incubated for an additional 48 h. Then both floating and adherent cells were collected and washed with PBS twice. Subsequently, the cells were fixed in 70% ethanol solution overnight and finally suspended in 50 μg/ml of PI solution containing 0.5% Triton X-100 and 2% RNase A. Finally, the cell cycle was determined using a CytoFLEX flow cytometer (Beckman Coulter, United States).

### Flow Cytometric Analysis of Apoptosis

Similar to the cell cycle analysis, U87MG and CHG-5 cells were plated in six-well plates (2 × 10^5^ cells/well), and treated with different concentrations (0, 25, 50, or 100 μM) of eriodictyol. After 48 h treatment, the cells were collected and washed with cold PBS and suspended in 100 μl of 1× binding buffer, followed by the addition of 5 μl of FITC Annexin V and 5 μl of PI and incubation for 10 min at room temperature in the dark. Finally, after the addition of 400 μl of 1× binding buffer, samples were analyzed with a CytoFLEX flow cytometer (Beckman Coulter).

### Hoechst 33342 Analysis

U87MG and CHG-5 cells were plated in 24-well plates at a density of 1 × 10^4^ cells per well. Different concentrations (0, 25, 50, or 100 μM) of eriodictyol were added to each well, and cells were incubated for 48 h. Subsequently, the cells were stained with Hoechst 33342 (10 μg/ml) for 10 min at 37°C in the dark. Subsequently, the cells were washed twice with PBS and observed by fluorescence microscopy at 200× magnification (Nikon).

### TUNEL Assay for Eriodictyol-Induced Apoptosis

U87MG and CHG-5 cells were seeded into 24-well plates (1 × 10^4^ cells/well) and incubated overnight. The cells were treated with different concentrations of eriodictyol for 48 h. Apoptosis was determined by TUNEL assay. The TUNEL assay was performed according to the instruction manual (Vazyme Biotech, China). Cells were photographed by fluorescence microscopy at 200× magnification (Nikon).

### Western Blot Analysis

Cells and tumor tissues were lysed in RIPA buffer containing 1 mM protease inhibitor and 1 mM phosphatase inhibitor (Bimake, Houston, TX, USA). The protein concentration was measured by bicinchoninic acid (BCA) assay (BCA kit, Beyotime Biotechnology, China). Subsequently, proteins were separated by (6%, 8%, and 12%) sodium dodecyl sulfate-polyacrylamide gel electrophoresis (SDS-PAGE), electrotransferred to polyvinylidene difluoride (PVDF) membranes (Millipore, Billerica, MA, USA), and incubated with primary antibodies overnight at 4°C. Subsequently, the PVDF membranes were washed with TBST for three times and then incubated with secondary antibodies for 2 h at room temperature. Finally, protein bands were visualized by an Odyssey^®^ CLx Imaging System (LI‐COR Biosciences, United States).

### Reverse Transcription Quantitative Polymerase Chain Reaction (RT-qPCR)

Briefly, total RNA was extracted following the instruction manual of the RNA extraction kit (TIANGEN Biotech, China), and 1 μg of total RNA was reverse transcribed to cDNA by using the All-in-One cDNA Synthesis SuperMix kit (Bimake). Then, qPCR was performed using the PCR kit according to the instructions. Expression values were normalized to the expression of β-actin. Primers used in this study were the following: Bax: 5′-TGCGTCCACCAAGAAGC-3′ (forward), 5′-TCCAGTTCGTCCCCGAT-3′ (reverse); Bcl-2: 5′-GCGGATTGACATTTCTGTG-3′ (forward), 5′-CATAAGGCAACGATCCCA-3′ (reverse); Bcl-xL: 5′-CCTGGGTTCCCTTTCCTT-3′ (forward), 5′-TCCTGGTCCTTGCATCTTT-3′ (reverse); β-actin: 5′-AGGGTGTTGTGGAGATGGG-3′ (forward), 5′-TGGCCTTGAGTTTCCTGCT-3′ (reverse).

### Immunohistochemistry Staining

Tissue sections were dewaxed, soaked in ethanol, and then blocked with 3% H_2_O_2_ for 10 min. Next, the tissue sections were washed carefully by distilled water and blocked with 5% goat serum. Then the sections were incubated with primary antibodies overnight at 4°C. Subsequently, sections were washed with PBS and further incubated with secondary antibodies. Finally, tissue sections were stained with DAB and observed with a light microscope at 200× magnification (Nikon).

### Xenograft Mouse Model

Nude mice (50% male and 50% female, 4–5 weeks old) were obtained from the Animal Ethics Committee of Chongqing Medical University and housed in a specific pathogen-free laboratory environment. The nude mice were injected subcutaneously with U87MG cells (5 × 10^6^) to establish the xenograft model. When tumors had grown to about 50 mm^3^, mice were divided randomly into five groups and injected intraperitoneally with normal saline, 50, 100, or 200 mg/kg eriodictyol, or 50 mg/kg temozolomide once per day for 21 days. Tumor size and mouse body weight were measured every three days. After three weeks, all mice were sacrificed, and the tumors were excised, weighed, and fixed in 4% paraformaldehyde for further analysis.

### Statistical Analysis

Statistical analysis was carried out using GraphPad 6.0 software. The results are presented as means ± SD or SEM. Data were analyzed using Student's *t*-test or ANOVA, and *P* < 0.05 was considered to indicate statistical significance.

## Results

### Eriodictyol Inhibits the Proliferation of Glioma Cells in Vitro

To evaluate the potential anti-cancer effect of eriodictyol on cancer cells, we treated several cancer cell lines (NCI-H1975 lung cancer, HCT116 colon cancer, CAL148 breast cancer, PANC1 pancreatic cancer, U87MG glioma, and HepG2 liver cancer cell lines) with different concentrations of eriodictyol (0, 25, 50, 100, 200, or 400 μM). After 48 h, the proliferation of cancer cell lines was examined through the CCK-8 assay. Our data demonstrate that eriodictyol could suppress cancer cell proliferation, especially in U87MG glioma cells (**Figure 1B**). Then, in order to further explore the anti-proliferation effect of eriodictyol on glioma cells, the CCK-8 assay was repeated with four glioma cell lines (U87MG, CHG-5, A172, and T98-G). The results are shown in **Figure 1C**. The growth of U87MG and CHG-5 glioma cells was significantly inhibited by eriodictyol treatment in a dose- and time-dependent manner (**Figures 1D, E**). IC_50_ values of eriodictyol for U87MG and CHG-5 cells were presented in **Table 1**. Moreover, the anti-proliferation effect of eriodictyol was strong on glioma cells but very weak on normal mouse astrocytes (**Figures 1F, G**).

**Table 1:** Eriodictyol IC_50_ values for glioma cell lines.

Cell line	IC_50_ (μM)		
	24 h	48 h	72 h
U87MG	190.63 ± 1.92	51.65 ± 1.28	36.10 ± 1.58
CHG-5	203.10 ± 2.13	114.19 ± 1.69	71.66 ± 1.11

IC_50_ is presented as mean ± SEM.

### Eriodictyol Inhibits Cell Colony Formation in U87MG and CHG-5 Cells

**Figure 2:**
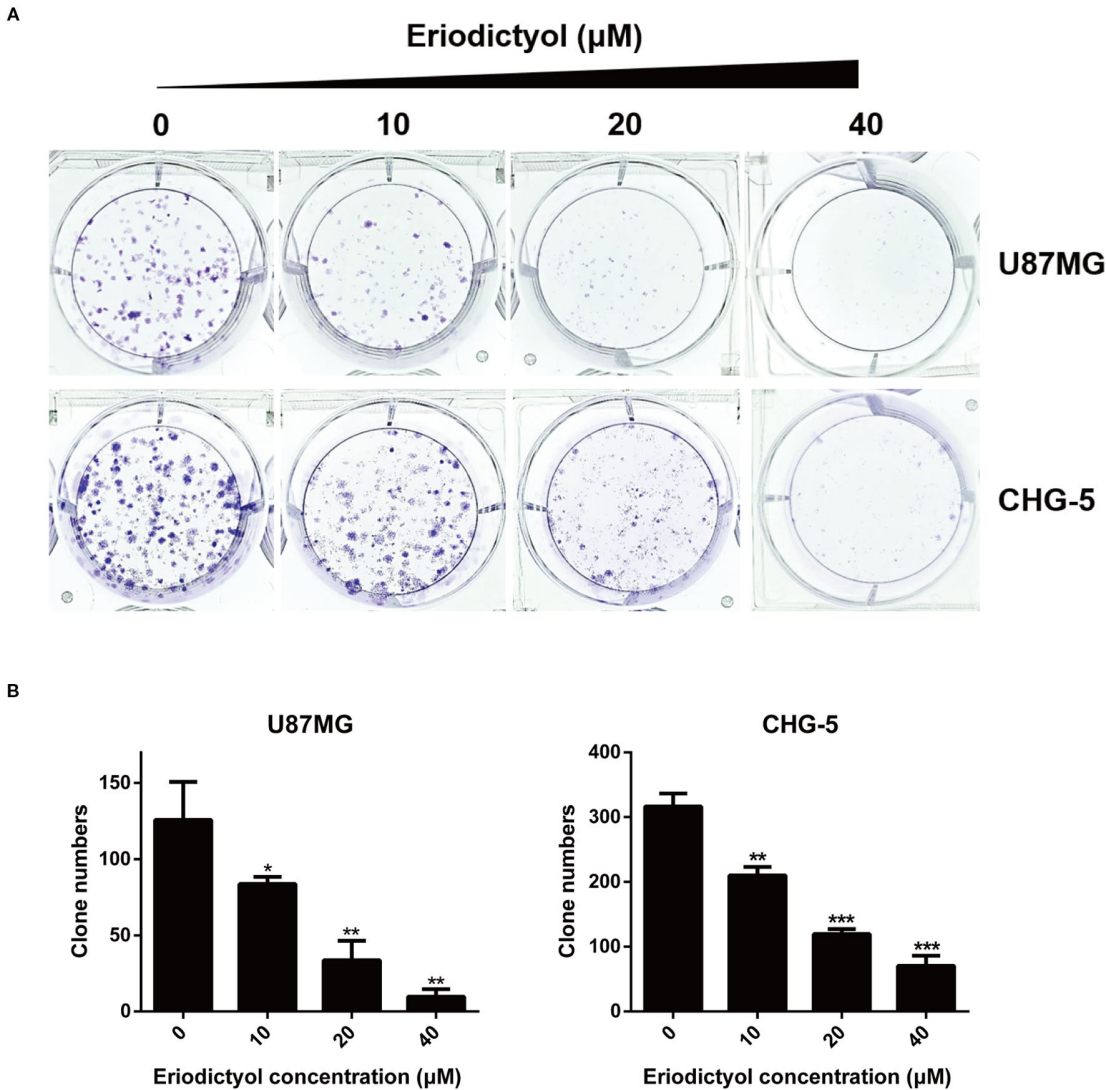
Effects of eriodictyol on the colony formation ability of glioma cells. **(A)** U87MG and CHG-5 cells were treated with eriodictyol (0, 10, 20, or 40 μM) for 12 days, and stained with 0.5% crystal violet solution. **(B)** Quantification of the number of colonies. The data are shown as the mean ± SD of three experiments. *P < 0.05, **P < 0.01, ***P < 0.001 compared with the control group.

U87MG and CHG-5 cells were seeded into six-well plates and cultured with different concentrations of eriodictyol (0, 10, 20, or 40 μM) for 12 days. As shown in **Figures 2A, B**, the colony formation ability of U87MG and CHG-5 cells was dramatically inhibited by eriodictyol.

### Eriodictyol Inhibits U87MG and CHG-5 Cell Migration and Invasion

**Figure 3:**
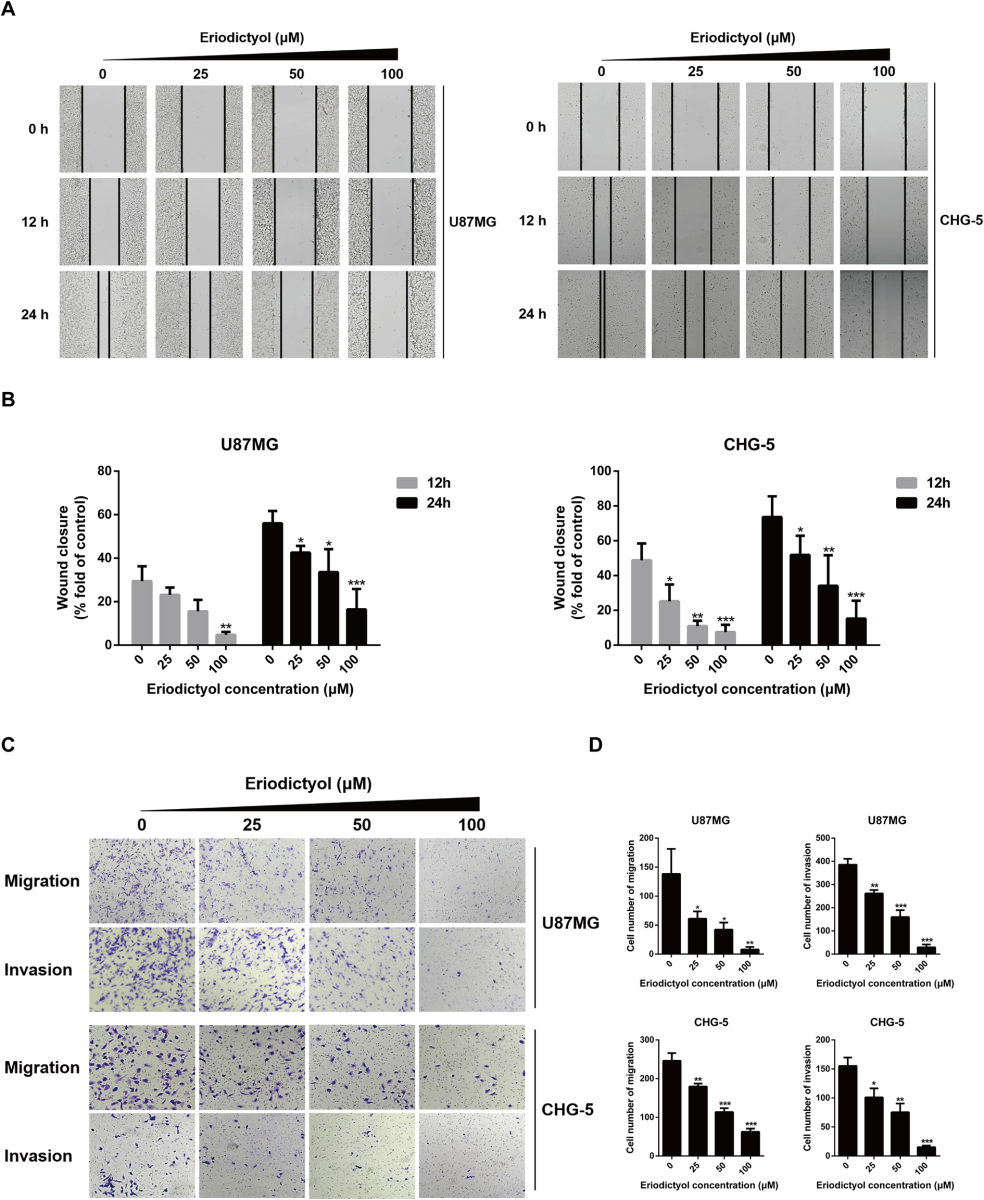
Eriodictyol inhibits the migration and invasion of U87MG and CHG-5 cells *in vitro*. **(A)** The cell migration ability was determined by wound healing assay. **(B)** Quantification of the migrated cells. **(C)** The cell migration and invasion were determined by Transwell assay. **(D)** Quantification of the migrated and invasive cells. The data are shown as the mean ± SD of three experiments. *P < 0.05, **P < 0.01, ***P < 0.001 compared with the control group.

The anti-migration and anti-invasion effects of eriodictyol on U87MG and CHG-5 cells were evaluated by wound healing and Transwell assays. Eriodictyol significantly inhibited the wound healing ability of U87MG and CHG-5 cells in a dose- and time-dependent manner (**Figures 3A, B**). Moreover, the Transwell assay showed that (i) eriodictyol markedly inhibited the migration ability of U87MG and CHG-5 cells, consistent with the wound healing assay, and (ii) the number of cells which passed through the membrane was obviously reduced with increasing eriodictyol concentrations (0, 25, 50, and 100 μM) (**Figures 3C, D**).

### Eriodictyol Induces Cell Cycle Arrest at the S Phase in U87MG and CHG-5 cells

**Figure 4:**
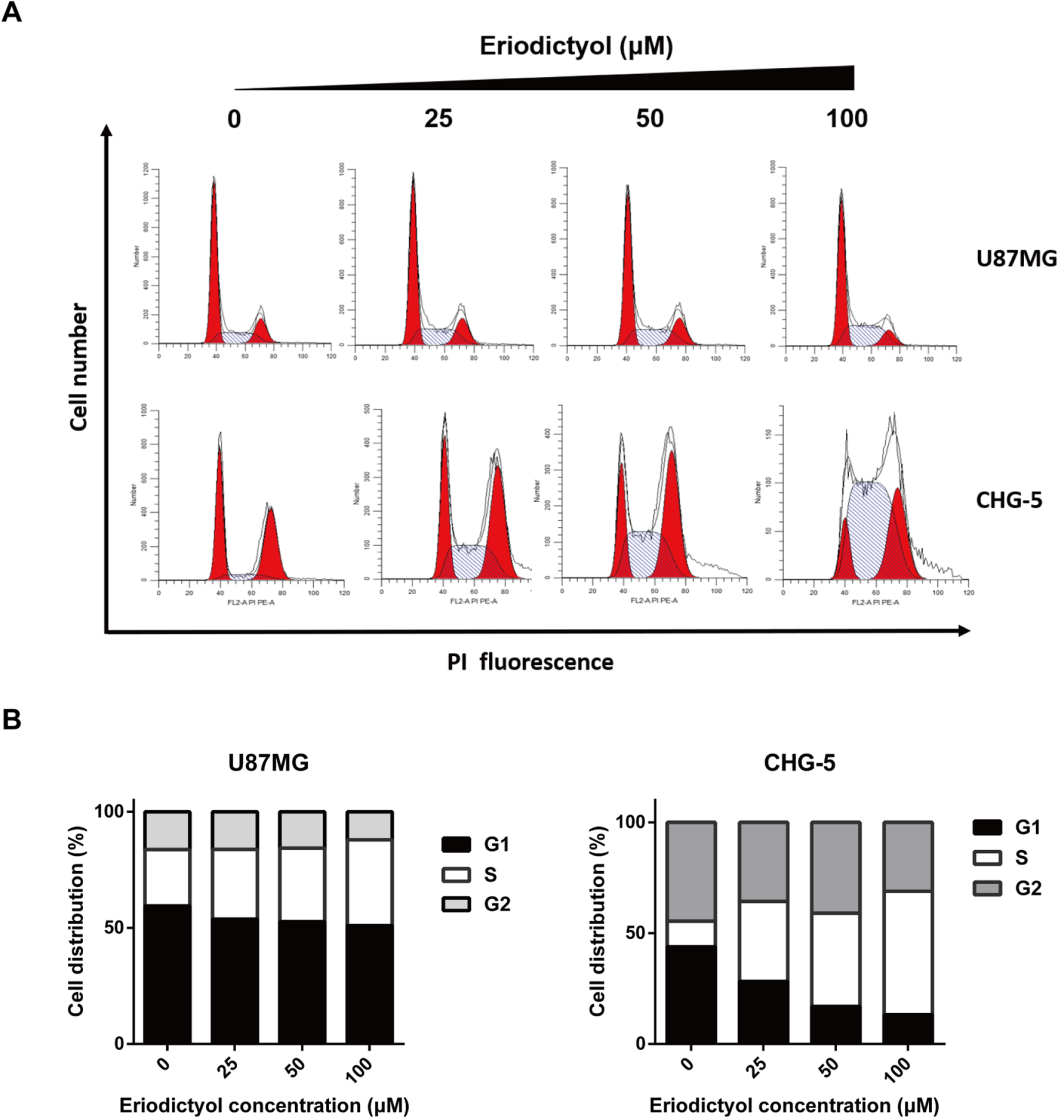
Eriodictyol induces cell cycle arrest in U87MG and CHG-5 cells. **(A)** U87MG and CHG-5 cells were treated with eriodictyol (0, 25, 50, or 100 μM) for 48 h, and cell cycle arrest was examined by flow cytometry. **(B)** Bar graphs showing the percentages of U87MG and CHG-5 cells in different phases.

To investigate the effects of eriodictyol on the cell cycle, we treated U87MG and CHG-5 cells with eriodictyol for 48 h, and their cell cycle status was determined by flow cytometry. The data indicate that eriodictyol arrests the cell cycle at the S phase (**Figures 4A, B**).

### Eriodictyol Significantly Induces Apoptosis in U87MG and CHG-5 cells

**Figure 5:**
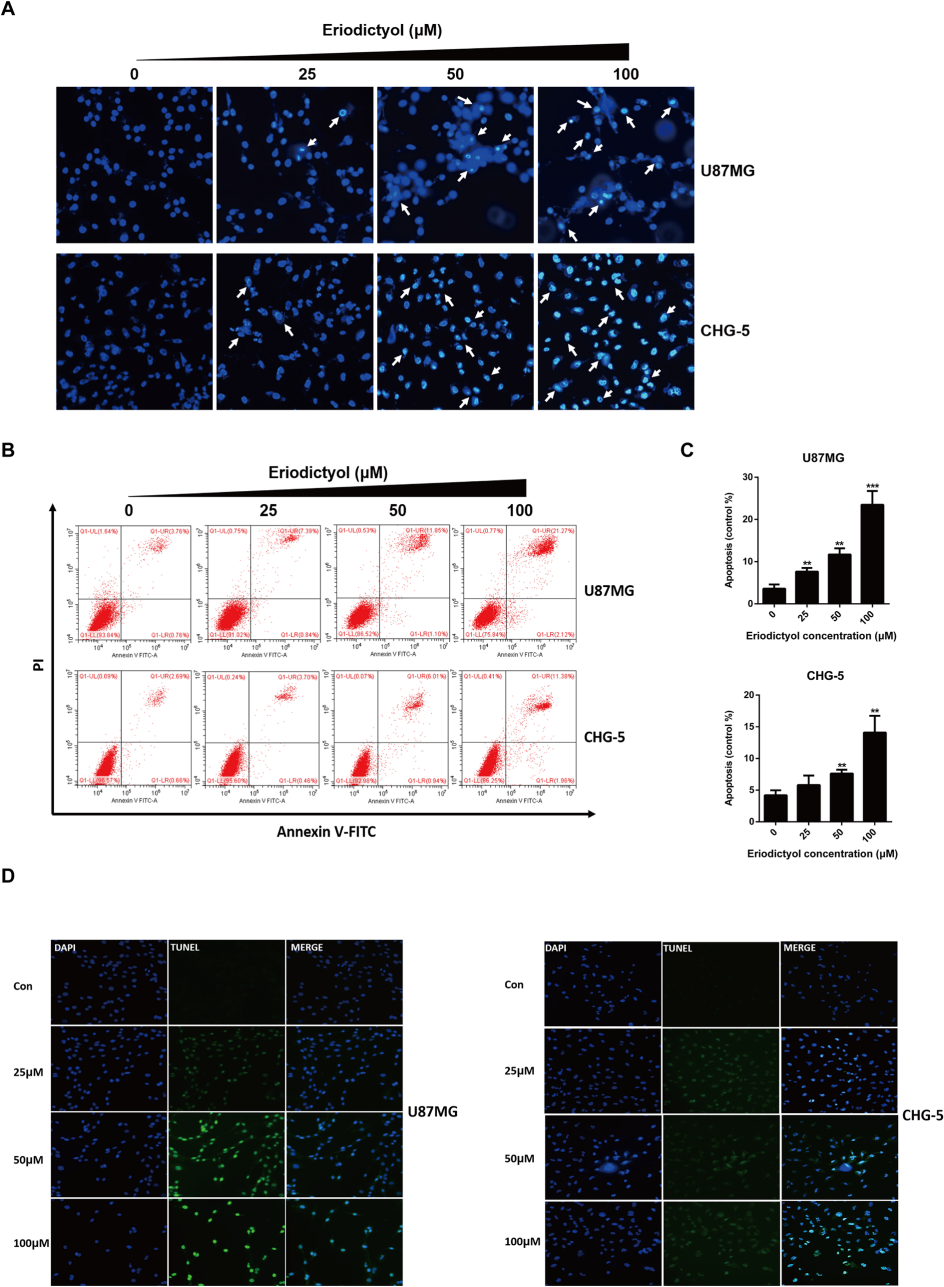
Eriodictyol induces apoptosis in U87MG and CHG-5 cells. **(A**, **B)** U87MG and CHG-5 were treated with eriodictyol (0, 25, 50, or 100 μM) for 48 h, and the effects of eriodictyol on apoptosis of glioma cells were determined by Hoechst 33342 analysis and flow cytometry. **(C)** Quantification of the apoptotic cells. **(D)** Cell apoptosis was detected by TUNEL assay. The data are shown as the mean ± SD of three experiments. *P < 0.05, **P < 0.01, ***P < 0.001 compared with the control group.

The effects of eriodictyol on glioma cell apoptosis were investigated by flow cytometry, Hoechst 33342 assay, and TUNEL assay. Firstly, the apoptotic effects on glioma cells (U87MG and CHG-5) were detected by Hoechst 33342 assay. The cell brightness, the degree of chromatin condensation, and nuclear fragmentation increased upon treatment with increasing eriodictyol concentrations (0, 25, 50, and 100 μM) (**Figure 5A**). Then, we used flow cytometry to further evaluate the anti-cancer effects of eriodictyol. As shown in **Figures 5B, C**, the number of apoptotic cells increased with increasing eriodictyol concentrations. Finally, we examined the apoptotic effects of eriodictyol by TUNEL assay. The fluorescence intensity of U87MG and CHG-5 increased upon eriodictyol treatment (**Figure 5D**).

### The Effects of Eriodictyol on the Expression of Apoptotic Markers

**Figure 6:**
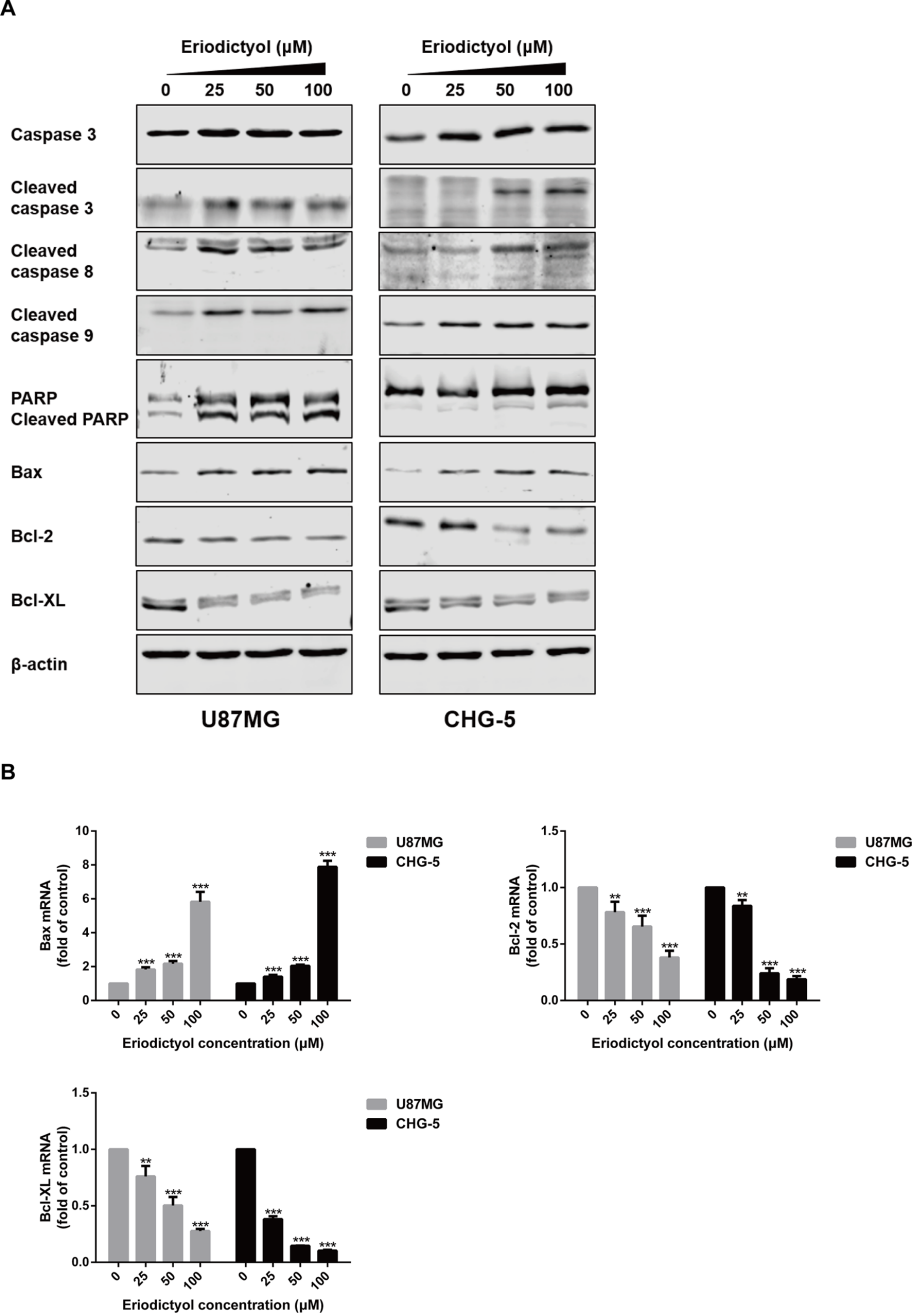
Effects of eriodictyol on the expression of apoptosis markers in glioma cells. **(A)** U87MG and CHG-5 cells were treated with eriodictyol (0, 25, 50, or 100 μM) for 48 h, and the expression levels of cell apoptosis-related proteins were measured by Western blot. **(B)** The mRNA levels of *Bax*, *Bcl-2*, and *Bcl-xL* in U87MG and CHG-5 cells were measured by RT-qPCR after treatment with eriodictyol for 48 h. The data are shown as the mean ± SD of three experiments. **P < 0.01, ***P < 0.001 compared with the control group.

To further explore the apoptotic effects of eriodictyol on U87MG and CHG-5 cells, expression levels of a series of apoptosis-related proteins were measured by Western blot. As shown in **Figure 6A**, the expression levels of cleaved Caspase-3, 8, and 9, cleaved PARP, and Bax in U87MG and CHG-5 cells were increased upon eriodictyol treatment, while the expression levels of anti-apoptotic proteins (Bcl-2 and Bcl-xL) were decreased. Next, we detected the mRNA levels of *Bax*, *Bcl-2*, and *Bcl-xL* in U87MG and CHG-5 cells after treatment with eriodictyol. The changes in mRNA levels were consistent with our Western blot results (**Figure 6B**).

### The Effects of Eriodictyol on the PI3K/Akt/NF-κB Signaling Pathway

**Figure 7:**
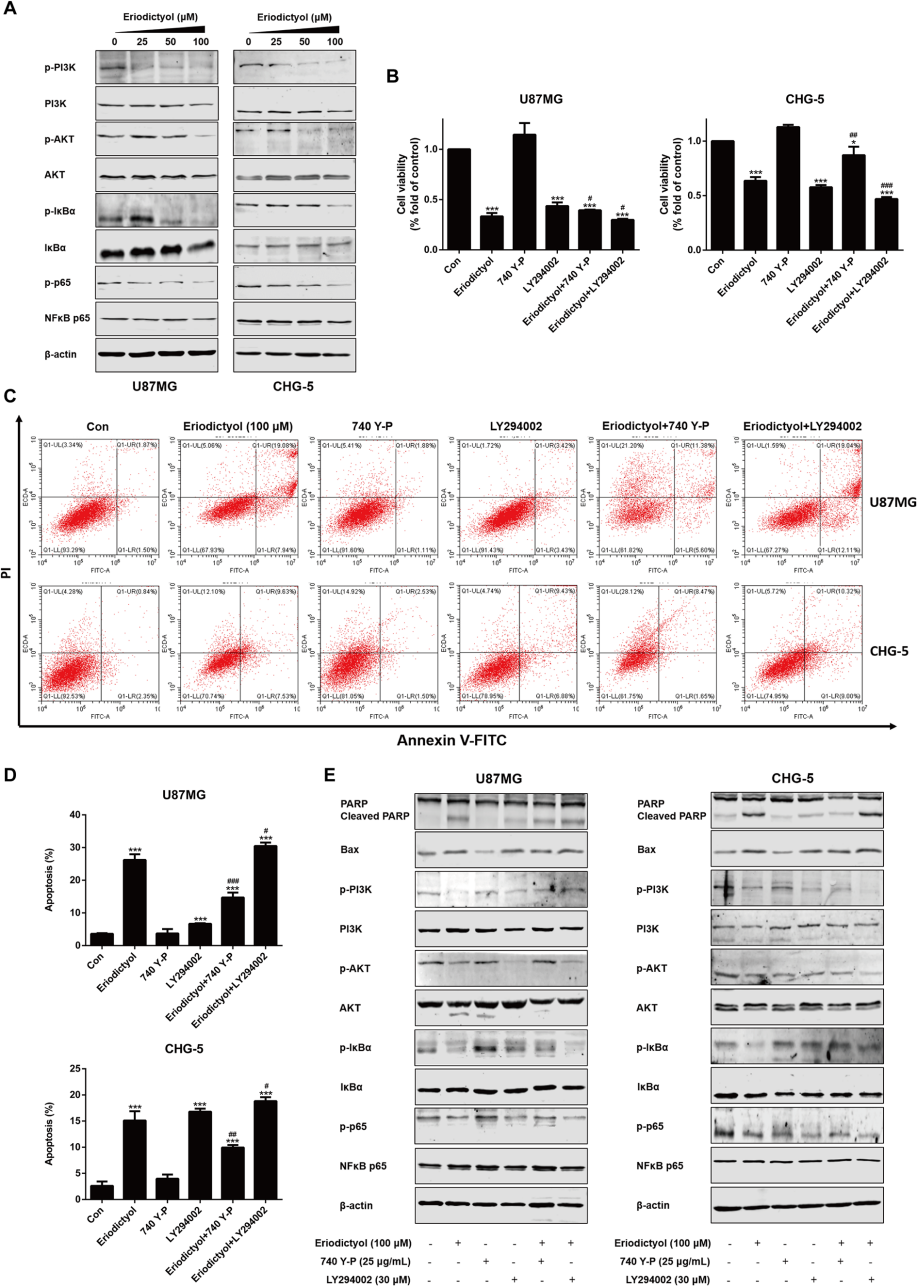
Eriodictyol-induced apoptosis of glioma cells is mediated by the PI3K/Akt/NF-κB signaling pathway. **(A)** Western blot analysis of the expression of PI3K/Akt/NF-κB signaling pathway proteins after treatment with eriodictyol (0, 25, 50, or 100 μM) for 48 h. **(B)** Viability of U87MG and CHG-5 cells as measured by CCK-8 assay. Cells were treated with or without eriodictyol (100 μM) for 48 h after PI3K agonist (740 Y-P, 25 μg/ml) or inhibitor (LY294002, 30 μM) pretreatment for 2 h. **(C)** The number of apoptotic cells was examined by flow cytometry after treatment as in **(B)**. **(D)** Quantification of the apoptotic cells. **(E)** Expression levels of Bax, PARP, and PI3K/Akt/NF-κB signaling pathway proteins were measured by Western blot after treatment as in **(B)**. The data are shown as the mean ± SD of three experiments. *P < 0.01, ***P < 0.001 compared with the control group; ^#^ *P* < 0.05, ^##^ *P* < 0.01, ^###^ *P* < 0.001 compared with the eriodictyol group.

To investigate the mechanisms by which eriodictyol induces apoptosis in glioma cells, we measured the levels of proteins in the PI3K/Akt/NF-κB signaling pathway by Western blot. The expression levels of p-PI3K, p-Akt, p-IκBα, and p-NF-κB were downregulated upon treatment with eriodictyol (**Figure 7A**).

### Activation of the PI3K/Akt/NF-κB Signaling Pathway Attenuates the Apoptotic Effects of Eriodictyol on U87MG and CHG-5 cells

To further investigate the mechanisms underlying eriodictyol-induced apoptosis in glioma cells, we measured cell viability by CCK-8 assay after treatment with eriodictyol, 740 Y-P (PI3K agonist), LY294002 (PI3K inhibitor), eriodictyol combined with 740 Y-P, or eriodictyol combined with LY294002. As shown in **Figure 7B**, eriodictyol significantly inhibited the cell viability of U87MG and CHG-5 cells. Moreover, 740 Y-P (25 μg/ml) reversed the eriodictyol-induced decline in cell viability, and LY294002 (30 μM) enhanced the effects of eriodictyol on cell viability. Next, we measured the rate of apoptotic cells by flow cytometry, and the results were consistent with our CCK-8 assay results (**Figures 7C, D**). The expression levels of apoptosis-related and PI3K/Akt/NF-κB signaling pathway proteins were measured by Western blot. The expression levels of Bax and cleaved PARP were increased by eriodictyol, while the opposite was observed for 740 Y-P (**Figure 7E**). Moreover, 740 Y-P significantly reversed the eriodictyol-inhibited phosphorylation of PI3K, Akt, IκBα, and NF-κB. Collectively, these results suggest that the eriodictyol-induced apoptosis of U87MG and CHG-5 cells might be correlated with the PI3K/Akt/NF-κB signaling pathway.

### Eriodictyol Inhibits Glioma Growth and Induced Apoptosis in Xenograft Mouse Model

**Figure 8:**
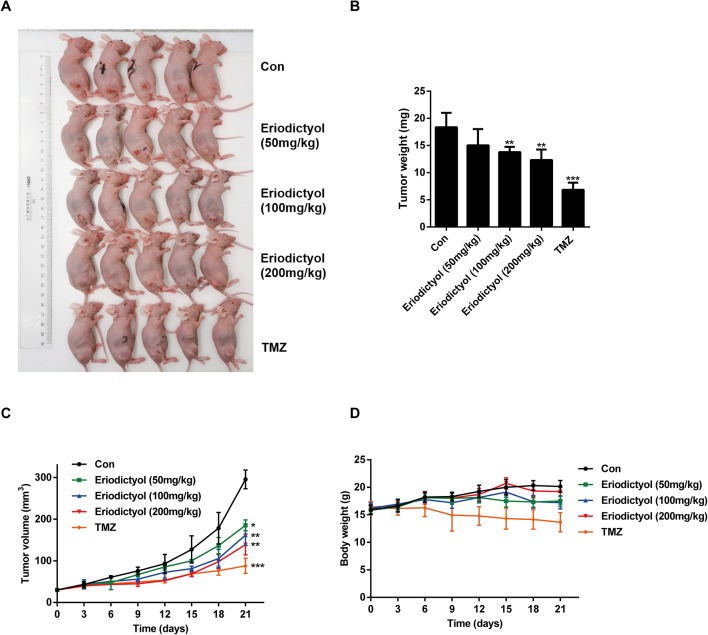
Eriodictyol inhibits glioma growth *in vivo*. **(A)** Nude mice were injected subcutaneously with U87MG cells to establish the xenograft model. When tumors had grown to about 50 mm^3^, mice were injected intraperitoneally with 50, 100, or 200 mg/kg eriodictyol or 50 mg/kg temozolomide once per day for 21 days. **(B)** The tumors were weighed at the end of the study. **(C**, **D)** Tumor volume and mouse body weight were measured every three days. All data are shown as the mean ± SD. *P < 0.05, **P < 0.01, ***P < 0.001 compared with the control group.

**Figure 9:**
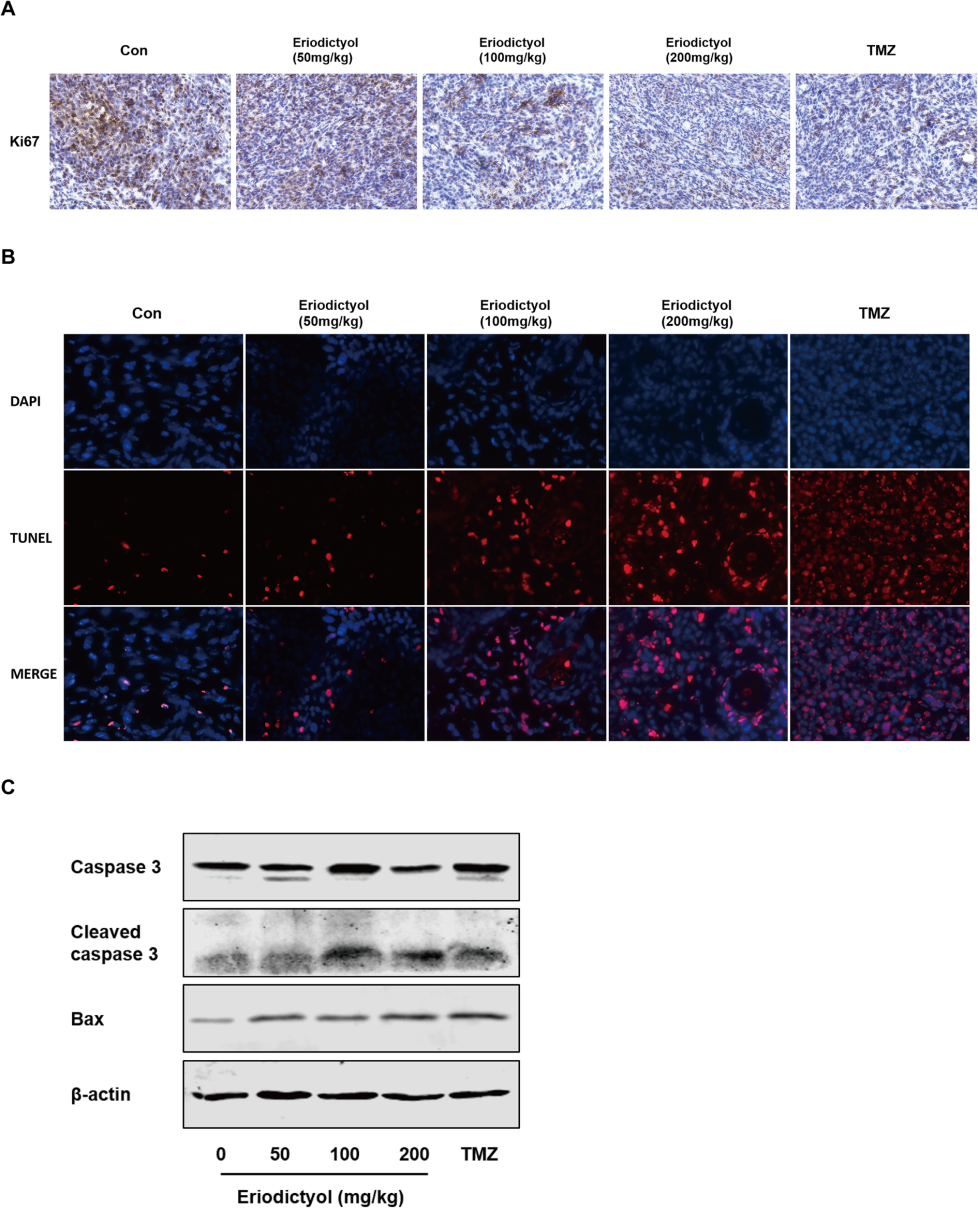
Eriodictyol inhibits proliferation and induces apoptosis of glioma cells *in vivo*. **(A**, **B)** Proliferation and apoptosis were examined in tumor tissue by Ki67 staining and TNUEL assay (magnification, 200×). **(C)** Expression levels of Caspase-3 and Bax in tumor tissue were measured by Western blot.

We also investigated the anti-cancer activities of eriodictyol *in vivo*. A xenograft mouse model was established using U87MG cells and nude mice (**Figure 8A**). We found that eriodictyol dose-dependently reduced the tumor volume and weight (**Figures 8B, C**). Furthermore, the weight loss of mice in the eriodictyol group was significantly smaller than that of the temozolomide group compared with the control group (**Figure 8D**). However, the anti-cancer effects of eriodictyol were weaker than those of temozolomide, even at high doses. After TUNEL staining, the number of TUNEL-positive cells in tumor tissue sections was increased after eriodictyol treatment. The number of Ki67-positive cells, however, decreased after eriodictyol treatment in a dose-dependent manner (**Figures 9A, B**). The expression levels in tumor tissue of Caspase-3 and Bax were measured by Western blot. As shown in **Figure 9C**, the expression levels of cleaved Caspase-3 and Bax were increased upon eriodictyol treatment. These results illustrate that eriodictyol could inhibit glioma growth *in vivo*.

## Discussion

Glioma is the most common and aggressive malignant tumor found in the CNS (Ostrom et al., 2017). Chemotherapy plays an important role in the treatment and prognosis of patients with glioma (Patil et al., 2013). However, the efficacy of current chemotherapies is not ideal due to side effects and the development of drug resistance. Therefore, the discovery of new drugs with more potency and less toxicity is essential.

Eriodictyol is a natural flavonoid compound. Previous studies have shown that eriodictyol exerts anti-inflammatory and antioxidant effects both *in vitro* and *in vivo* (Li et al., 2018; He et al., 2019). However, the effects of eriodictyol on cancer and its underlying mechanisms largely remain unexplored.

In the present study, we have investigated the anti-cancer effects of eriodictyol in human glioma cells *in vitro* and *in vivo*. Our results demonstrate that eriodictyol can significantly suppress proliferation, metastasis and induce apoptosis in glioma cells. First, we examined the anti-cancer activity of eriodictyol in various cancer cell lines, including lung, colon, breast, pancreas, liver cancer and glioma cell lines. In addition, we evaluated the cytotoxicity of eriodictyol on normal mouse astrocytes. Interestingly, we found that eriodictyol could inhibit the cell viability of many cancer cell lines, especially glioma cells, but slightly of normal mouse astrocytes, indicating that eriodictyol has high anti-tumor activity and low toxicity. Second, we assessed the effects of eriodictyol on the proliferation of glioma cells by colony formation assay. The results demonstrate that eriodictyol could significantly inhibit colony formation of glioma cells in a dose-dependent manner. The high invasiveness and fast migration of gliomas is one of the main reasons for poor prognosis in patients with glioma (Qi et al., 2017). Therefore, to improve the prognosis of patients with glioma, it is important to effectively inhibit invasion and migration of glioma. We evaluated the effects of eriodictyol on invasion and migration of glioma cells by wound healing and Transwell assays. The data show that eriodictyol markedly suppressed invasion and migration of human glioma cells. Moreover, we found that the cell cycle was arrested at the S phase upon eriodictyol treatment. This suggests that eriodictyol might induce DNA replication damage in glioma cells (Bailon-Moscoso et al., 2017). Similar to our findings, other anti-cancer flavonoid compounds, such as ampelopsin, quercetin, and baicalein also arrest the cell cycle at the S phase (Mu et al., 2016; Srivastava et al., 2016; Cheng et al., 2017).

Apoptosis, one of the main ways of cell death, plays a critical role in homeostasis in human bodies (Rubinstein and Kimchi, 2012). Induction of apoptosis in tumors is a key mechanism targeted by many anti-cancer drugs (Jiang et al., 2015). In this study, the effects of eriodictyol on apoptosis in glioma cells were detected by flow cytometry, Hoechst 33342 assay, and TUNEL assay. We observed that eriodictyol could markedly induce apoptosis in U87MG and CHG-5 glioma cells. Moreover, we also found that eriodictyol induced apoptosis in U87MG and CHG-5 cells by activating Caspase-3, 8, 9 and cleaving PARP. We further investigated the Bcl-2 protein family and discovered that the expression of Bax was increased and that of Bcl-2 and Bcl-xL was decreased after eriodictyol treatment. According to their roles in the regulation of apoptosis, the Bcl-2 family proteins can be divided into two groups, i.e., Bax, Bad, Bid, etc., which promote apoptosis, and Bcl-2 and Bcl-xL, which inhibit apoptosis. Upregulation of Bax and downregulation of Bcl-2 and Bcl-xL leads to (i) the release of cytochrome C from mitochondria, (ii) the activation of mitochondrion-dependent Caspase, and hence (iii) the induction of apoptosis in cells (Pena-Blanco and Garcia-Saez, 2018).

PI3K/Akt signaling plays a critical role in cellular proliferation, survival, and metabolism (Yang et al., 2016). The expression of PI3K/Akt signaling pathway proteins is aberrantly upregulated in a variety of cancers (Garcia-Echeverria and Sellers, 2008). Activation of the PI3K signaling pathway can promote cancer cell proliferation and suppress cancer cell death (Janku et al., 2018). The NF-κB signaling pathway is one of the most important signaling pathways regulating cellular inflammatory responses. In addition, NF-κB signaling also plays critical roles in cancer development (Hoesel and Schmid, 2013). Upregulation of the NF-κB signaling pathway, which induces NF-κB translocation from the cytoplasm to the nucleus, could promote cancer metastasis (Perkins, 2012). Therefore, targeting of the PI3K/Akt/NF-κB signaling pathway might be a promising strategy to treat cancer.

Zhang et al. reported that eriodictyol could increase insulin-stimulated glucose uptake in human hepatocellular carcinoma cells by regulating the PI3K/Akt signaling pathway (Zhang et al., 2012). In addition, eriodictyol was found to reduce nitric oxide, TNF-α, IL-6, and IL-1β production in LPS-stimulated RAW264.7 cells through blocking the MAPK/NF-κB signaling pathway (Lee, 2011). Therefore, we hypothesized that the mechanisms underlying the anti-glioma effects of eriodictyol might be mediated through regulation of the PI3K/Akt/NF-κB signaling pathway. The present study indicates that eriodictyol dose-dependently induces apoptosis through inhibiting the phosphorylation of PI3K, Akt, and NF-κB. To further verify whether the PI3K/Akt/NF-κB signaling pathway plays a key role in eriodictyol-elicited apoptosis, the PI3K agonist 740 Y-P and he PI3K inhibitor LY294002 were employed. The agonist 740 Y-P significantly reversed the eriodictyol-induced apoptosis. In addition, 740 Y-P also reversed the eriodictyol-mediated PI3K/Akt/NF-κB inactivation, Bax upregulation, and cleaved PARP upregulation. LY294002 promoted eriodictyol-induced cell apoptosis and PI3K/Akt/NF-κB inhibition. These results imply that eriodictyol-induced apoptosis of U87MG and CHG-5 cells is mediated, at last partially, *via* the PI3K/Akt/NF-κB signaling pathway.

Finally, we evaluated the anti-tumor effects of eriodictyol *in vivo*. In the xenograft mouse model, eriodictyol induced apoptosis of glioma cells and inhibited tumor growth in a dose-dependent manner. Ki67 staining showed that eriodictyol effectively inhibited tumor growth. Both TUNEL staining and Western blot demonstrated that eriodictyol could induce apoptosis in tumor cells *in vivo*. Moreover, the weight loss of mice in the eriodictyol group was significantly smaller than that in the temozolomide group, and insignificantly lower than in the control group, indicating that eriodictyol has lower toxicity than temozolomide. However, the anti-cancer effects of eriodictyol *in vivo* were weaker than those of temozolomide, even at high doses. This problem might be solved by modifying the structure of eriodictyol to enhance its anti-cancer activity. According to *in vitro* and *in vivo* studies, eriodictyol could be developed as a promising therapeutic agent for glioma. Our research is a preliminary study on the anti-cancer effect of eriodictyol. In the future, we will further investigate the mechanisms underlying the anti-glioma effects presented in the present study, and we will try to modify the structure of eriodictyol to improve its anti-glioma activity.

## Conclusion

Our current study illustrates that the natural flavonoid eriodictyol could effectively inhibit glioma growth, possibly by inducing apoptosis through blocking the PI3K/Akt/NF-κB signaling pathway. These findings provide evidence that eriodictyol is a potential therapeutic agent to treat glioma patients in the future.

## Data Availability Statement

The datasets generated for this study are available on request to the corresponding author.

## Ethics Statement

The animal study was reviewed and approved by the Animal Ethics Committee of Chongqing Medical University.

## Author Contributions

WL and SL designed the experiments. SL supervised the whole project. WL performed most experiments and wrote the paper. XL and XZ provided their professional guidance. FL, XX, QD, JY and GH provided technical support.

## Funding

This research was supported by the Development Project from Chongqing Science Technology Commission (cstc2014yykfA110023).

## Conflict of Interest

The authors declare that the research was conducted in the absence of any commercial or financial relationships that could be construed as a potential conflict of interest.

## References

[B1] Ahmad A. Biersack B. Li Y. Kong D. Bao B. Schobert R. (2013). Targeted regulation of PI3K/Akt/mTOR/NF-kappaB signaling by indole compounds and their derivatives: mechanistic details and biological implications for cancer therapy. Anticancer Agents Med. Chem. 13 (7), 1002–1013. 10.2174/18715206113139990078 23272910 PMC3901097

[B2] Alexander B. M. Cloughesy T. F. (2017). Adult Glioblastoma. J. Clin. Oncol. 35 (21), 2402–2409. 10.1200/JCO.2017.73.0119 28640706

[B3] Bailon-Moscoso N. Cevallos-Solorzano G. Romero-Benavides J. C. Orellana M. I. (2017). Natural compounds as modulators of cell cycle arrest: application for anticancer chemotherapies. Curr. Genomics 18 (2), 106–131. 10.2174/1389202917666160808125645 28367072 PMC5345333

[B4] Cheng P. Gui C. Huang J. Xia Y. Fang Y. Da G. (2017). Molecular mechanisms of ampelopsin from Ampelopsis megalophylla induces apoptosis in HeLa cells. Oncol. Lett. 14 (3), 2691–2698. 10.3892/ol.2017.6520 28928812 PMC5588129

[B5] Efferth T. Saeed M. E. M. Kadioglu O. Seo E. J. Shirooie S. Mbaveng A. T. (2019). Collateral sensitivity of natural products in drug-resistant cancer cells. Biotechnol. Adv. 38, 107342. 10.1016/j.biotechadv.2019.01.009 30708024

[B6] Garcia-Echeverria C. Sellers W. R. (2008). Drug discovery approaches targeting the PI3K/Akt pathway in cancer. Oncogene 27 (41), 5511–5526. 10.1038/onc.2008.246 18794885

[B7] He P. Yan S. Zheng J. Gao Y. Zhang S. Liu Z. (2018). Eriodictyol Attenuates LPS-Induced Neuroinflammation, Amyloidogenesis, and Cognitive Impairments *via the* Inhibition of NF-kappaB in Male C57BL/6J Mice and BV2 Microglial Cells. J. Agric. Food Chem. 66 (39), 10205–10214. 10.1021/acs.jafc.8b03731 30208700

[B8] He P. Yan S. Wen X. Zhang S. Liu Z. Liu X. (2019). Eriodictyol alleviates lipopolysaccharide-triggered oxidative stress and synaptic dysfunctions in BV-2 microglial cells and mouse brain. J. Cell Biochem. 120 (9), 14756–14770. 10.1002/jcb.28736 31016762

[B9] Hoesel B. Schmid J. A. (2013). The complexity of NF-κB signaling in inflammation and cancer. Mol. Cancer 12 (1), 86. 10.1186/1476-4598-12-86 23915189 PMC3750319

[B10] Janku F. Yap T. A. Meric-Bernstam F. (2018). Targeting the PI3K pathway in cancer: are we making headway? Nat. Rev. Clin. Oncol. 15 (5), 273–291. 10.1038/nrclinonc.2018.28 29508857

[B11] Jiang Q. L. Zhang S. Tian M. Zhang S. Y. Xie T. Chen D. Y. (2015). Plant lectins, from ancient sugar-binding proteins to emerging anti-cancer drugs in apoptosis and autophagy. Cell Prolif. 48 (1), 17–28. 10.1111/cpr.12155 25488051 PMC6496769

[B12] Kwon E. Y. Choi M. S. (2019). Dietary eriodictyol alleviates adiposity, hepatic steatosis, insulin resistance, and inflammation in diet-induced obese mice. Int. J. Mol. Sci. 20 (5), 1227. 10.3390/ijms20051227 PMC6429409 30862092

[B13] Lapointe S. Perry A. Butowski N. A. (2018). Primary brain tumours in adults. Lancet 392 (10145), 432–446. 10.1016/s0140-6736(18)30990-5 30060998

[B14] Lee J. K. (2011). Anti-inflammatory effects of eriodictyol in lipopolysaccharide-stimulated raw 264.7 murine macrophages. Arch. Pharm. Res. 34 (4), 671–679. 10.1007/s12272-011-0418-3 21544733

[B15] Li D. Lu N. Han J. Chen X. Hao W. Xu W. (2018). Eriodictyol attenuates myocardial ischemia-reperfusion injury through the activation of JAK2. Front. Pharmacol. 9, 33. 10.3389/fphar.2018.00033 29441020 PMC5797583

[B16] Liu Y. Yan X. (2019). Eriodictyol inhibits survival and inflammatory responses and promotes apoptosis in rheumatoid arthritis fibroblast-like synoviocytes through AKT/FOXO1 signaling. J. Cell Biochem. 120 (9), 14628–14635. 10.1002/jcb.28724 31009103

[B17] Lv P. Yu J. Xu X. Lu T. Xu F. (2019). Eriodictyol inhibits high glucose-induced oxidative stress and inflammation in retinal ganglial cells. J. Cell Biochem. 120 (4), 5644–5651. 10.1002/jcb.27848 30317656

[B18] Minato K.-i. Miyake Y. Fukumoto S. Yamamoto K. Kato Y. Shimomura Y. (2003). Lemon flavonoid, eriocitrin, suppresses exercise-induced oxidative damage in rat liver. Life Sci. 72 (14), 1609–1616. 10.1016/s0024-3205(02)02443-8 12551749

[B19] Mu J. Liu T. Jiang L. Wu X. Cao Y. Li M. (2016). The Traditional Chinese Medicine Baicalein Potently Inhibits Gastric Cancer Cells. J. Cancer 7 (4), 453–461. 10.7150/jca.13548 26918059 PMC4749366

[B20] Ostrom Q. T. Gittleman H. Liao P. Vecchione-Koval T. Wolinsky Y. Kruchko C. (2017). CBTRUS Statistical Report: Primary brain and other central nervous system tumors diagnosed in the United States in 2010-2014. Neuro Oncol. 19 (suppl_5), v1–v88. 10.1093/neuonc/nox158 29117289 PMC5693142

[B21] Patil S. A. Amira H. A. Jones T. S. Renukadevi P. Pfeffer L. M. Miller D. D. (2013). Novel approaches to glioma drug design and drug screening. Expert Opin. Drug Discovery 8 (9), 1135–1151. 10.1517/17460441.2013.807248 23738794

[B22] Pena-Blanco A. Garcia-Saez A. J. (2018). Bax, Bak and beyond - mitochondrial performance in apoptosis. FEBS J. 285 (3), 416–431. 10.1111/febs.14186 28755482

[B23] Perkins N. D. (2012). The diverse and complex roles of NF-kappaB subunits in cancer. Nat. Rev. Cancer 12 (2), 121–132. 10.1038/nrc3204 22257950

[B24] Qi X. T. Zhan J. S. Xiao L. M. Li L. Xu H. X. Fu Z. B. (2017). The unwanted cell migration in the brain: glioma metastasis. Neurochem. Res. 42 (6), 1847–1863. 10.1007/s11064-017-2272-2 28478595

[B25] Rubinstein A. D. Kimchi A. (2012). Life in the balance - a mechanistic view of the crosstalk between autophagy and apoptosis. J. Cell Sci. 125 (Pt 22), 5259–5268. 10.1242/jcs.115865 23377657

[B26] Srivastava S. Somasagara R. R. Hegde M. Nishana M. Tadi S. K. Srivastava M. (2016). Quercetin, a natural flavonoid interacts with DNA, arrests cell cycle and causes tumor regression by activating mitochondrial pathway of Apoptosis. Sci. Rep. 6, 24049. 10.1038/srep24049 27068577 PMC4828642

[B27] Stupp R. Mason W. P. van den Bent M. J. Weller M. Fisher B. Taphoorn M. J. B. (2005). Radiotherapy plus concomitant and adjuvant temozolomide for glioblastoma. New Engl. J. Med. 352 (10), 987–996. 10.1056/NEJMoa043330 15758009

[B28] Xie G. Meng X. Wang F. Bao Y. Huo J. (2017). Eriodictyol attenuates arsenic trioxide-induced liver injury by activation of Nrf2. Oncotarget 8 (40), 68668–68674. 10.18632/oncotarget.19822 28978146 PMC5620286

[B29] Yang S. X. Polley E. Lipkowitz S. (2016). New insights on PI3K/AKT pathway alterations and clinical outcomes in breast cancer. Cancer Treat Rev. 45, 87–96. 10.1016/j.ctrv.2016.03.004 26995633 PMC7436195

[B30] Zeng B. Chen K. Du P. Wang S. S. Ren B. Ren Y. L. (2016). Phenolic compounds from Clinopodium chinense (Benth.) O. Kuntze and their inhibitory effects on alpha-Glucosidase and vascular Endothelial cells injury. Chem. Biodivers. 13 (5), 596–601. 10.1002/cbdv.201500187 27088891

[B31] Zhang W. Y. Lee J. J. Kim Y. Kim I. S. Han J. H. Lee S. G. (2012). Effect of eriodictyol on glucose uptake and insulin resistance *in vitro* . J. Agric. Food Chem. 60 (31), 7652–7658. 10.1021/jf300601z 22809065

